# Poly[[bis­(μ_2_-6-methyl­pyrazin-2-carboxyl­ato-κ^3^ *N* ^1^,*O*:*N* ^4^)copper(II)] dihydrate]

**DOI:** 10.1107/S1600536809041440

**Published:** 2009-10-23

**Authors:** Chuan-gang Fan, Xin-Ting Wei, Linwei Li

**Affiliations:** a Department of Chemistry and Chemical Engineering, Binzhou University, Shandong 256603, People’s Republic of China; b College of Chemistry and Chemical Engineering, Liaocheng University, Shandong 252059, People’s Republic of China

## Abstract

In the title compound, {[Cu(C_6_H_5_N_2_O_2_)_2_]·2H_2_O}_*n*_, the Cu^II^ ion (site symmetry 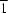) is coordinated by two *N*,*O*-bidentate ligands and two *N*-monodentate ligands in a distorted CuO_2_N_4_ octa­hedral geometry. Each anion acts as a bridge between two cations, thus forming a two-dimensional polymeric network parallel to the *ab* plane. The packing is consolidated by O—H⋯O hydrogen bonds. One of the O atoms of the ligand and both water mol­ecules are disordered.

## Related literature

For a related structure, see: Yigit *et al.* (2006 ▶). For background to coordination networks, see: Kesanli & Lin (2003 ▶); Barnett & Champness (2003 ▶).
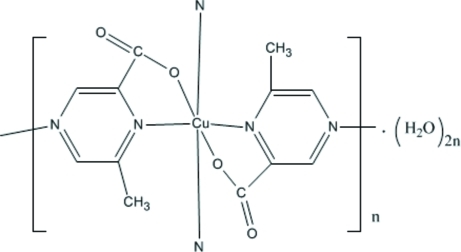


## Experimental

### 

#### Crystal data

[Cu(C_6_H_5_N_2_O_2_)_2_]·2H_2_O
*M* *_r_* = 373.81
Monoclinic, 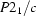

*a* = 8.371 (1) Å
*b* = 9.7901 (11) Å
*c* = 10.3849 (13) Å
β = 112.277 (1)°
*V* = 787.55 (16) Å^3^
*Z* = 2
Mo *K*α radiation
μ = 1.42 mm^−1^
*T* = 298 K
0.34 × 0.32 × 0.30 mm

#### Data collection

Bruker SMART CCD diffractometer
Absorption correction: multi-scan (*SADABS*; Bruker, 2003 ▶) *T*_min_ = 0.644, *T*_max_ = 0.675
3821 measured reflections
1388 independent reflections
1094 reflections with *I* > 2σ(*I*)
*R*_int_ = 0.027

#### Refinement

*R*[*F* ^2^ > 2σ(*F* ^2^)] = 0.049
*wR*(*F* ^2^) = 0.160
*S* = 1.06
1388 reflections
126 parameters
H-atom parameters constrained
Δρ_max_ = 1.23 e Å^−3^
Δρ_min_ = −0.47 e Å^−3^

### 

Data collection: *SMART* (Bruker, 2003 ▶); cell refinement: *SAINT* (Bruker, 2003 ▶); data reduction: *SAINT*; program(s) used to solve structure: *SHELXS97* (Sheldrick, 2008 ▶); program(s) used to refine structure: *SHELXL97* (Sheldrick, 2008 ▶); molecular graphics: *SHELXTL* (Sheldrick, 2008 ▶); software used to prepare material for publication: *SHELXTL*.

## Supplementary Material

Crystal structure: contains datablocks I, global. DOI: 10.1107/S1600536809041440/hb5123sup1.cif

Structure factors: contains datablocks I. DOI: 10.1107/S1600536809041440/hb5123Isup2.hkl

Additional supplementary materials:  crystallographic information; 3D view; checkCIF report

## Figures and Tables

**Table 1:** Selected bond lengths (Å)

Cu1—O1	1.949 (3)
Cu1—N2^i^	2.064 (4)
Cu1—N1	2.354 (4)

Symmetry code: (i) 
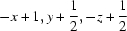
.

**Table 2:** Hydrogen-bond geometry (Å, °)

*D*—H⋯*A*	*D*—H	H⋯*A*	*D*⋯*A*	*D*—H⋯*A*
O3—H3*C*⋯O2^ii^	0.85	1.78	2.624 (3)	175
O3—H3*C*⋯O2′^ii^	0.85	2.36	3.181 (3)	163
O3—H3*D*⋯O2′^i^	0.85	2.23	3.074 (2)	175
O4—H4*D*⋯O3^iii^	0.85	1.97	2.73 (3)	147
O4—H4*E*⋯O3^iv^	0.85	2.01	2.77 (2)	150

Symmetry codes: (i) 
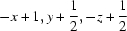
; (ii) 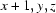; (iii) 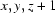; (iv) 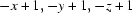.

**Fig. 1.**
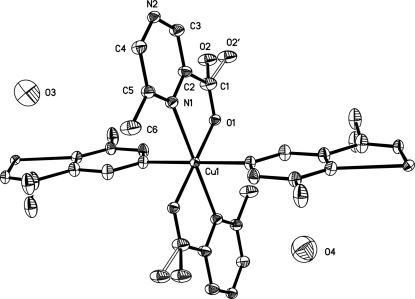
The molecular structure of (I) showing 30% probability displacement ellipsoids.

**Fig. 2.**
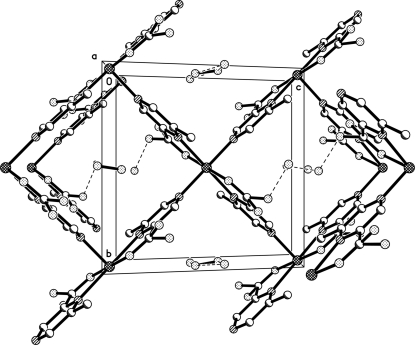
The crystal packing of (I), viewed approximately along the c axis.

## supplementary crystallographic information

### Comment

In recent years,the construction of metal-organic coordination polymers (MOCPs) by metal-directed self- assembly is of great interest not only for their potential applications as functional materials in ion exchange, catalysis, hydrogen storage, and magnetic devices, but also for their aesthetic structural and host–guest chemistry associated with large central cavities. (Kesanli *et al.*, 2003; Barnett *et al.*, 2003). As an extension of this work,the 6-methyl-2- pyrazinecarboxylic acid was chosen due to its chelating coordinated effect leading to a linear metal center.

As shown in Fig.1. X-ray structural analyses of complex (1) reveal the core structure of (1) is the symmetric dinuclear unit of [Cu(*L*)2(H_2_O)_2_] (*L* = anion of 6-methyl-2- pyrazinecarboxylic acid). Each Cu^II^ atom is coordinated to two ligands as well as to the two N atoms of the other two ligands, forming a octahedral coordination conformation.

The ligand forms a coordination polymer with tridentate and monodentate binding of Cu^II^ ions at opposite ends of the ligand bridge neighboring units and yield a two-dimensional arrangement running in the *ab* plane (Fig. 2). Yigit *et al.* (2006) recently reported a coordination polymer featuring a similar head-to-tail arrangement of pyrazine-2,3,5,6-tetracarboxylic acid. There is intermolecular H-bonds (O3—H3···O4) showed as strong H bond, which link the molecular and water to form host–guest model.

### Experimental

4.00 g Potassium permanganate was to be dissolved in 30 ml pure water in a beaker and 1.0 ml 98% H_2_SO_4_ was added to the solution. After stiring 10 min, and then added 2,6-dimethylpyrazine to the mixture.The recation was keeping at room temperature for 24 h. The resulting solution was filtered, and the filtrate was left in a beaker, then 2.00 g copper(II) sulfate pentahydrate was added to the filtrate. After stirrng for 20 min s, the copper(II) sulfate pentahydrate was solved completely. The blue solution was kept at the room temperature for two weeks and blue blocks of (I) were obtained. Yield: 86percent, m.p. 551 K. Anal. Calc. for C_12_H_14_CuN_4_O_6_: C: 38.5567; H:3.7750; N:14.9881; Found:*C*: 37.67; H: 3.86; N: 14.32%. Selected IR (KBr, cm-1) 3439(w), 2889 (w), 1638(*s*), 1596 (*s*), 1536(w), 1409(*s*), 1364(*m*), 1275(*m*), 1149(*s*), 1151(*s*), 1034(*s*), 941(*s*), 820(*m*), 800(*s*), 712(w), 531(*m*), 471(w).

### Refinement

All H atoms were placed geometrically and treated as riding on their parent atoms with C—H 0.93(pyrazine), C—H 0.97 (methylene) Å [*U*_iso_(H) = 1.2*U*_eq_(C)] and O—H 0.82 Å (hydroxyl) [*U*_iso_(H) = 1.5*U*_eq_(O)].

### Crystal data

**Table:**

[Cu(C_6_H_5_N_2_O_2_)_2_]·2H_2_O	*F*(000) = 382
*M**_r_* = 373.81	*D*_x_ = 1.576 Mg m^−^^3^
Monoclinic, *P*2_1_/*c*	Mo *K*α radiation, λ = 0.71073 Å
Hall symbol: -P 2ybc	Cell parameters from 1598 reflections
*a* = 8.371 (1) Å	θ = 2.6–26.2°
*b* = 9.7901 (11) Å	µ = 1.42 mm^−^^1^
*c* = 10.3849 (13) Å	*T* = 298 K
β = 112.277 (1)°	Block, blue
*V* = 787.55 (16) Å^3^	0.34 × 0.32 × 0.30 mm
*Z* = 2	

### Data collection

**Table:**

Bruker SMART CCD diffractometer	1388 independent reflections
Radiation source: fine-focus sealed tube	1094 reflections with *I* > 2σ(*I*)
graphite	*R*_int_ = 0.027
ω scans	θ_max_ = 25.0°, θ_min_ = 2.6°
Absorption correction: multi-scan (*SADABS*; Bruker, 2003)	*h* = −9→9
*T*_min_ = 0.644, *T*_max_ = 0.675	*k* = −11→11
3821 measured reflections	*l* = −12→9

### Refinement

**Table:**

Refinement on *F*^2^	Primary atom site location: structure-invariant direct methods
Least-squares matrix: full	Secondary atom site location: difference Fourier map
*R*[*F*^2^ > 2σ(*F*^2^)] = 0.049	Hydrogen site location: inferred from neighbouring sites
*wR*(*F*^2^) = 0.160	H-atom parameters constrained
*S* = 1.06	*w* = 1/[σ^2^(*F*_o_^2^) + (0.0987*P*)^2^ + 1.8186*P*] where *P* = (*F*_o_^2^ + 2*F*_c_^2^)/3
1388 reflections	(Δ/σ)_max_ = 0.001
126 parameters	Δρ_max_ = 1.23 e Å^−^^3^
0 restraints	Δρ_min_ = −0.47 e Å^−^^3^

### Special details

**Table:**

Geometry. All e.s.d.'s (except the e.s.d. in the dihedral angle between two l.s. planes) are estimated using the full covariance matrix. The cell e.s.d.'s are taken into account individually in the estimation of e.s.d.'s in distances, angles and torsion angles; correlations between e.s.d.'s in cell parameters are only used when they are defined by crystal symmetry. An approximate (isotropic) treatment of cell e.s.d.'s is used for estimating e.s.d.'s involving l.s. planes.
Refinement. Refinement of *F*^2^ against ALL reflections. The weighted *R*-factor *wR* and goodness of fit *S* are based on *F*^2^, conventional *R*-factors *R* are based on *F*, with *F* set to zero for negative *F*^2^. The threshold expression of *F*^2^ > σ(*F*^2^) is used only for calculating *R*-factors(gt) *etc*. and is not relevant to the choice of reflections for refinement. *R*-factors based on *F*^2^ are statistically about twice as large as those based on *F*, and *R*- factors based on ALL data will be even larger.

### Fractional atomic coordinates and isotropic or equivalent isotropic displacement parameters (Å^2^)

**Table:**

	*x*	*y*	*z*	*U*_iso_*/*U*_eq_	Occ. (<1)
Cu1	0.5000	0.5000	0.5000	0.0210 (3)	
N1	0.5565 (5)	0.3429 (4)	0.3509 (4)	0.0273 (9)	
N2	0.5246 (5)	0.1511 (4)	0.1450 (4)	0.0270 (9)	
O1	0.2704 (4)	0.4333 (3)	0.3861 (3)	0.0259 (7)	
O2	0.1014 (18)	0.343 (6)	0.184 (5)	0.049 (10)	0.46 (9)
O2'	0.1110 (16)	0.279 (5)	0.235 (4)	0.049 (9)	0.54 (9)
O3	0.8445 (18)	0.5171 (12)	0.0914 (16)	0.097 (4)	0.50
H3C	0.9307	0.4639	0.1248	0.116*	0.50
H3D	0.8631	0.5877	0.1426	0.116*	0.50
O4	0.499 (3)	0.4908 (16)	0.9398 (18)	0.126 (6)	0.50
H4D	0.5904	0.5025	1.0121	0.152*	0.50
H4E	0.4122	0.4929	0.9628	0.152*	0.50
C1	0.2469 (6)	0.3484 (6)	0.2899 (6)	0.0411 (14)	
C2	0.4033 (6)	0.2942 (5)	0.2678 (5)	0.0302 (11)	
C3	0.3863 (6)	0.1987 (6)	0.1653 (5)	0.0337 (12)	
H3	0.2771	0.1672	0.1096	0.040*	
C4	0.6768 (6)	0.1996 (5)	0.2266 (5)	0.0319 (11)	
H4A	0.7750	0.1682	0.2146	0.038*	
C5	0.6955 (6)	0.2969 (5)	0.3308 (5)	0.0301 (11)	
C6	0.8676 (7)	0.3506 (7)	0.4211 (6)	0.0496 (16)	
H6A	0.8532	0.4283	0.4721	0.074*	
H6B	0.9300	0.3775	0.3644	0.074*	
H6C	0.9309	0.2809	0.4850	0.074*	

### Atomic displacement parameters (Å^2^)

**Table:**

	*U*^11^	*U*^22^	*U*^33^	*U*^12^	*U*^13^	*U*^23^
Cu1	0.0214 (5)	0.0226 (5)	0.0193 (5)	−0.0019 (3)	0.0079 (3)	0.0006 (3)
N1	0.023 (2)	0.033 (2)	0.026 (2)	−0.0023 (17)	0.0093 (16)	−0.0047 (17)
N2	0.025 (2)	0.031 (2)	0.027 (2)	−0.0020 (18)	0.0120 (16)	−0.0056 (17)
O1	0.0234 (16)	0.0301 (18)	0.0267 (17)	−0.0018 (14)	0.0124 (13)	−0.0062 (15)
O2	0.025 (5)	0.065 (19)	0.054 (13)	−0.004 (6)	0.012 (5)	−0.030 (15)
O2'	0.025 (4)	0.065 (17)	0.054 (11)	−0.004 (5)	0.012 (5)	−0.030 (12)
O3	0.074 (8)	0.092 (10)	0.115 (11)	0.018 (6)	0.026 (8)	0.013 (7)
O4	0.120 (14)	0.133 (15)	0.129 (17)	−0.002 (9)	0.050 (14)	0.000 (11)
C1	0.019 (3)	0.058 (4)	0.045 (3)	−0.004 (2)	0.010 (2)	−0.019 (3)
C2	0.025 (3)	0.033 (3)	0.032 (3)	−0.004 (2)	0.010 (2)	−0.010 (2)
C3	0.025 (3)	0.041 (3)	0.035 (3)	−0.004 (2)	0.011 (2)	−0.012 (2)
C4	0.023 (3)	0.040 (3)	0.034 (3)	0.002 (2)	0.012 (2)	−0.007 (2)
C5	0.026 (3)	0.032 (3)	0.033 (3)	−0.002 (2)	0.012 (2)	−0.006 (2)
C6	0.024 (3)	0.068 (4)	0.054 (4)	−0.001 (3)	0.011 (3)	−0.027 (3)

### Geometric parameters (Å, °)

**Table:**

Cu1—O1^i^	1.949 (3)	O3—H3C	0.8500
Cu1—O1	1.949 (3)	O3—H3D	0.8500
Cu1—N2^ii^	2.064 (4)	O4—O4^v^	1.26 (3)
Cu1—N2^iii^	2.064 (4)	O4—H4D	0.8500
Cu1—N1^i^	2.354 (4)	O4—H4E	0.8500
Cu1—N1	2.354 (4)	C1—C2	1.509 (7)
N1—C2	1.333 (6)	C2—C3	1.383 (7)
N1—C5	1.336 (6)	C3—H3	0.9300
N2—C4	1.322 (6)	C4—C5	1.405 (7)
N2—C3	1.337 (6)	C4—H4A	0.9300
N2—Cu1^iv^	2.065 (4)	C5—C6	1.486 (7)
O1—C1	1.256 (6)	C6—H6A	0.9600
O2—C1	1.30 (3)	C6—H6B	0.9600
O2'—C1	1.26 (2)	C6—H6C	0.9600
			
O1^i^—Cu1—O1	180.0	H4D—O4—H4E	109.0
O1^i^—Cu1—N2^ii^	89.70 (14)	O1—C1—O2'	124.0 (8)
O1—Cu1—N2^ii^	90.30 (14)	O1—C1—O2	121.0 (11)
O1^i^—Cu1—N2^iii^	90.30 (14)	O2'—C1—O2	36.7 (6)
O1—Cu1—N2^iii^	89.70 (14)	O1—C1—C2	118.1 (4)
N2^ii^—Cu1—N2^iii^	180.0	O2'—C1—C2	115.3 (8)
O1^i^—Cu1—N1^i^	77.27 (13)	O2—C1—C2	116.5 (8)
O1—Cu1—N1^i^	102.73 (13)	N1—C2—C3	122.2 (5)
N2^ii^—Cu1—N1^i^	88.74 (15)	N1—C2—C1	116.9 (4)
N2^iii^—Cu1—N1^i^	91.26 (15)	C3—C2—C1	120.9 (4)
O1^i^—Cu1—N1	102.73 (13)	N2—C3—C2	121.0 (4)
O1—Cu1—N1	77.27 (13)	N2—C3—H3	119.5
N2^ii^—Cu1—N1	91.26 (15)	C2—C3—H3	119.5
N2^iii^—Cu1—N1	88.74 (15)	N2—C4—C5	122.4 (4)
N1^i^—Cu1—N1	180.0	N2—C4—H4A	118.8
C2—N1—C5	117.4 (4)	C5—C4—H4A	118.8
C2—N1—Cu1	106.0 (3)	N1—C5—C4	120.0 (4)
C5—N1—Cu1	136.6 (3)	N1—C5—C6	118.4 (4)
C4—N2—C3	117.1 (4)	C4—C5—C6	121.6 (4)
C4—N2—Cu1^iv^	122.0 (3)	C5—C6—H6A	109.5
C3—N2—Cu1^iv^	120.8 (3)	C5—C6—H6B	109.5
C1—O1—Cu1	121.7 (3)	H6A—C6—H6B	109.5
H3C—O3—H3D	108.5	C5—C6—H6C	109.5
O4^v^—O4—H4D	55.8	H6A—C6—H6C	109.5
O4^v^—O4—H4E	53.3	H6B—C6—H6C	109.5
			
O1^i^—Cu1—N1—C2	−179.1 (3)	C5—N1—C2—C1	−179.1 (5)
O1—Cu1—N1—C2	0.9 (3)	Cu1—N1—C2—C1	0.0 (5)
N2^ii^—Cu1—N1—C2	90.9 (3)	O1—C1—C2—N1	−1.4 (8)
N2^iii^—Cu1—N1—C2	−89.1 (3)	O2'—C1—C2—N1	−164 (3)
N1^i^—Cu1—N1—C2	168 (100)	O2—C1—C2—N1	155 (4)
O1^i^—Cu1—N1—C5	−0.4 (5)	O1—C1—C2—C3	178.9 (5)
O1—Cu1—N1—C5	179.6 (5)	O2'—C1—C2—C3	17 (3)
N2^ii^—Cu1—N1—C5	−90.4 (5)	O2—C1—C2—C3	−24 (4)
N2^iii^—Cu1—N1—C5	89.6 (5)	C4—N2—C3—C2	−0.2 (8)
N1^i^—Cu1—N1—C5	−13 (100)	Cu1^iv^—N2—C3—C2	176.2 (4)
O1^i^—Cu1—O1—C1	5(100)	N1—C2—C3—N2	−0.1 (8)
N2^ii^—Cu1—O1—C1	−93.0 (4)	C1—C2—C3—N2	179.6 (5)
N2^iii^—Cu1—O1—C1	87.0 (4)	C3—N2—C4—C5	0.0 (8)
N1^i^—Cu1—O1—C1	178.2 (4)	Cu1^iv^—N2—C4—C5	−176.3 (4)
N1—Cu1—O1—C1	−1.8 (4)	C2—N1—C5—C4	−0.8 (7)
Cu1—O1—C1—O2'	163 (3)	Cu1—N1—C5—C4	−179.5 (4)
Cu1—O1—C1—O2	−153 (4)	C2—N1—C5—C6	179.4 (5)
Cu1—O1—C1—C2	2.4 (7)	Cu1—N1—C5—C6	0.7 (8)
C5—N1—C2—C3	0.6 (7)	N2—C4—C5—N1	0.5 (8)
Cu1—N1—C2—C3	179.7 (4)	N2—C4—C5—C6	−179.7 (5)

Symmetry codes: (i) −*x*+1, −*y*+1, −*z*+1; (ii) *x*, −*y*+1/2, *z*+1/2; (iii) −*x*+1, *y*+1/2, −*z*+1/2; (iv) −*x*+1, *y*−1/2, −*z*+1/2; (v) −*x*+1, −*y*+1, −*z*+2.

### Hydrogen-bond geometry (Å, °)

**Table:**

*D*—H···*A*	*D*—H	H···*A*	*D*···*A*	*D*—H···*A*
O3—H3C···O2^vi^	0.85	1.78	2.624 (3)	175
O3—H3C···O2'^vi^	0.85	2.36	3.181 (3)	163
O3—H3D···O2'^iii^	0.85	2.23	3.074 (2)	175
O4—H4D···O3^vii^	0.85	1.97	2.73 (3)	147
O4—H4E···O3^i^	0.85	2.01	2.77 (2)	150

Symmetry codes: (vi) *x*+1, *y*, *z*; (iii) −*x*+1, *y*+1/2, −*z*+1/2; (vii) *x*, *y*, *z*+1; (i) −*x*+1, −*y*+1, −*z*+1.
